# Prostate-Related Germline Variant Frequencies Detected in a Cohort of Men With Metastatic Prostate Cancer in Northern India

**DOI:** 10.1200/PO-25-00130

**Published:** 2025-07-10

**Authors:** Atul Batra, Jessica G. Cockburn, Abhenil Mittal, Rui M. Bernardino, Tiiu Sildva, Marian Severin Wettstein, Amlesh Seth, Brusabhanu Nayak, Sameer Bakhshi, Ranjit K. Sahoo, Akash Kumar, Rishabh Jain, Seema Kaushal, Mayank Singh, Sneha Gund, Sunakshi Chowdhary, Karina Lakhani, Krishna Patel, Raymond H. Kim, Mohammad R. Akbari, Neil Eric Fleshner

**Affiliations:** ^1^ Department of Medical Oncology, Dr BRA-IRCH, All India Institute of Medical Sciences (AIIMS), New Delhi, India; ^2^ Department of Surgical Oncology, Princess Margaret Cancer Centre, University Health Network, Toronto, ON; ^3^ Department of Oncology, Health Sciences North, Northern Ontario School of Medicine, Sudbury, ON; ^4^ Department of Urology, All India Institute of Medical Sciences (AIIMS), New Delhi, India; ^5^ Department of Pathology, All India Institute of Medical Sciences (AIIMS), New Delhi, India; ^6^ Division of Medical Oncology and Hematology, Princess Margaret Cancer Centre, University Health Network, Toronto, ON; ^7^ Women's College Research and Innovation Center, Women's College Hospital, University of Toronto, Toronto, ON; ^8^ Dalla Lana School of Public Health, University of Toronto, Toronto, ON; ^9^ Department of Urologic Oncology, Princess Margaret Cancer Centre, University Health Network, Toronto, ON

## Abstract

**PURPOSE:**

Although prostate cancer is generally associated with favorable outcomes, metastatic disease remains incurable. Additionally, a subset of individuals with high-risk or metastatic disease are likely to harbor at least one germline variant in known prostate cancer association genes. Because of differences in cohort selection and sequencing strategies, the prevalence of germline variants in global populations is unclear.

**METHODS:**

A whole-exome sequencing (WES) approach was used to explore germline variants in a cohort of patients with metastatic prostate cancer from India. In total, 276 individuals treated at the All India Institute of Medical Sciences in New Delhi, India, were prospectively and consecutively recruited. Blood specimens underwent standard WES and bioinformatic analysis to determine the prevalence of pathogenic and likely pathogenic (PV/LPV) prostate cancer variants, which were then assessed for associations with clinical features.

**RESULTS:**

In total, PV/LPVs were detected in 11% of individuals across eight genes linked to prostate cancer, most frequently in BRCA2 (3.98%). The distribution reflects previously published findings from other global cohorts, although frequencies in the prevalence of specific variants differ slightly. No relationship between variant status and clinical features were detected, although analysis of a larger cohort may show otherwise.

**CONCLUSION:**

These results indicate that germline screening for prostate cancer following existing guidelines yield similar variant detection frequencies when focusing on individuals with metastatic disease in the Indian context. In summary, some men are more likely to develop an advanced form of metastatic prostate cancer than others because of differences in their genes, known as variants. This study looked at the how many of these variants are in a group of patients from India. We found that the number of variants in this group was similar to those from other parts of the world, including more found in a gene called *BRCA2*.

## INTRODUCTION

Individuals who develop high-risk or metastatic prostate cancer (PCa) are increasingly being recognized for harboring higher frequencies of germline pathogenic (PV) or likely pathogenic (LPV) germline variants. Although evidence continues to emerge, the prevalence of PV/LPV is estimated to be between 6% and 17% of cases worldwide, most of which are found in genes relating to the homologous recombination repair pathway (HRR).^1^ *BRCA2* is the most frequently mutated gene across prostate cancer cohorts, with lower but consistent detection of variations found in *ATM*, *CHEK2*, *BRCA1*, *RAD51D*, and *PALB2* among others^2–13^ (Table 1). Other cancer susceptibility genes have also been identified (Data Supplement, Table S1), although most have yet to be contributory for prostate cancer specifically.

**CONTEXT**

**Key Objective**
To compare pathogenic and likely pathogenic variant (PV/LPV) detection frequency in a cohort of patients with metastatic prostate cancer (PCa) from India with those previously reported in cohorts from other geographical regions.
**Knowledge Generated**
Of the 19 genes included in the National Comprehensive Cancer Network (NCCN) genetic testing guidelines, PV/LPVs were detected in eight from patients in this cohort of patients with metastatic disease. The overall distribution of PV/LPVs detected in this cohort from India is most similar to reports from European-focused cohorts, but variation in specific gene variants is evident.
**Relevance**
These findings support the use of NCCN guidelines in India, although the evolving landscape of PCa genetics may help to refine inclusion criteria and genes tested.

**TABLE 1.** Description of Global Cohorts

Citation	Location	Inclusion Criteria	Cohort Size	Study Design	Sequencing Strategy	No. of Genes Analyzed and Variant Frequency (%)
Chávarri-Guerra et al^4^	Mexico	Patients diagnosed with prostate cancer who meet NCCN criteria (3)	199	Prospective cohort	Panel	108 (2)
Song et al^5^	South Korea	Cases: Patients with mPC unselected for family history of cancer (5) Controls: Healthy (no cancer)	340:495	Case/control	WGS	20 (8.8)
Saunders et al^6^	European	Final data set was enriched for patients with aggressive, fatal, and younger-onset disease, in addition to an FH of PrCa	6,805	Pooled retrospective cohorts	WES or panel	10 (8.1)
Coelho et al^7^	Brazil	Men with primary PCa (early- and late-onset) (7)	71	Retrospective cohort	WES or panel	20 (9.86)
Momozawa et al^8^	Japan	Unselected PCa patients (10)	7,636: 12,366	Case/control	Targeted sequencing	8 (2.9)
Pritchard et al^9^	United Kingdom/United States	Patients with metastatic PCa who were unselected for family history of cancer or age at diagnosis (11)	692	Prospective cohort	Targeted sequencing	20 (11.8)
Nicolosi et al^10^	Various	Patients with PCa who underwent genetic testing	3,607	Retrospective cohort	Panels (varied)	14 (17.20)
Wei et al^11^	Chinese	Unselected for family history or age at diagnosis; includes regional and distant metastases, NCCN high risk and low-intermediate risk	316	Retrospective cohort	Panel sequencing	18 (9.8)
Park et al^12^	South Korea	Patients with metastatic PCa, unselected for family history of cancer or age	301	Prospective cohort	Panel sequencing	Approximately 100 (12)
Cheng et al^13^	United States	Patients with metastatic PCa, unselected for family history of cancer	551	Prospective cohort	Panel sequencing	30 (8.7)

Abbreviations: FH, family history; mPC, metastatic prostate cancer; NCCN, National Comprehensive Cancer Network; PCa, prostate cancer; WES, whole-exome sequencing; WGS, whole-genome sequencing.

Differences in global distribution of germline variants may be related to several factors. For example, variant frequencies of PV/LPVs as well as variant of unknown significance reflect the genetic diversity of each cohort,^14–16^ which may have limited representation. Also, regional differences in health care practices, such as routine screening, affect disease prevalence and severity, which may confound detection of certain variants associated with clinical features.^4,5,9^ Furthermore, reported variant frequencies are based on a range of study designs, including the use of broad versus targeted sequencing approaches and analysis of selected genes of interest. So, although a consensus exists about the pathogenic nature of certain variants in predisposing men to PCa, more work is needed to determine a universal set of clinically relevant genes.

Understanding the etiology of PCa through a genetics lens provides an opportunity for screening and early detection that can mitigate the consequences of high-risk disease. To do this effectively, it is necessary to have a comprehensive understanding of variants that contribute to disease risk in different populations, particularly given that frequency of stage at diagnoses can vary greatly between countries.^17^ As such, there is a need to thoroughly assess the PV/LPVs reflected in different regions, particularly those that are not adequately captured in existing reports (Table 1). This will help to refine genetic testing guidelines for PCa that can be applied globally. Furthermore, genotype can be used to guide treatment selection, as PARP inhibitors have enhanced efficacy in those with *BRCA1* and *BRCA2* variants.^18–21^

India comprises a large population with a unique demography of PCa and has consistently higher rates of advanced-stage diagnoses, begging inquiry about the frequency of germline PV/LPVs, which have yet to be reported. While there is limited information, PCa incidence in India is relatively lower than in Western countries, reports have shown that between 41.6% and 50% and upwards of 85% of PCa diagnoses have been associated with late-stage disease.^22–24^ Interestingly, higher prevalence of PV/LPVs have been detected in patients with breast cancer from India, where there is both a higher prevalence of more aggressive histologies (eg, triple-negative breast cancer).^25^ As such, it is reasonable to expect a unique genomic profile in Indian patients with metastatic PCa. The primary goal of this work is to characterize the prevalence of established germline variants related to PCa in patients with metastatic PCa in India using a prospective cohort and whole-exome sequencing (WES). These findings will help to guide genetic testing practices that can support screening for susceptibility to high-risk PCa.

## METHODS

### Study Setting

This study was conducted at the All India Institute of Medical Sciences, New Delhi, which serves the Northern region of India, including the capital city, Delhi, and the neighboring states. The institute has two cancer blocks, (1) Dr Bhimrao Ambedkar Institute Rotary Cancer Hospital and (2) the National Cancer Institute, which collectively register and treat approximately 15,000 patients with cancer annually. The study was approved a priori by the Institute Ethics Committees at both institutions, and each patient provided informed consent before participating in this study.

### Patient Population

The patient population reflects individuals diagnosed with de novo metastatic PCa treated at a large tertiary referral public hospital in India, including those representing a range of socioeconomic groups. Prospective recruitment of patients with metastatic disease, namely those presenting with metastatic PCa as determined by any evidence of distant metastasis on prostate-specific membrane antigen-positron emission tomography or computed tomography scans from either of the two cancer blocks, was conducted between April 2022 and December 2023.

### Demographic and Clinical Variables

Clinical data from patient charts were abstracted to confirm eligibility, eligibility assessment, and downstream descriptive and statistical analysis. Specifically, variables were collected that pertained to age at diagnosis, baseline prostate-specific antigen (PSA), pathologic variables (grade group and staging), as well as volume and location of metastases. Tumor-related variables were used to classify risk and tumor volume on the basis of definitions provided by the CHAARTED and LATITUDE trials.^26,27^

### Whole-Exome Sequencing

High-quality genomic DNA was extracted from blood samples using the QIAamp DNA Blood kit (QIAGEN, New Delhi, India). After the prelibrary quality assessment, samples with a required quantity of minimum 100 ng of DNA were selected for the WES library preparation. The precapture DNA libraries were prepared using the xGen DNA Library Prep EZ kit (Integrated DNA Technologies [IDT], Coralville, IA), following the manufacturer's protocol. The precapture libraries underwent QC checks including library quantification and library size distribution. Next, 500 ng of each sample's QC-validated precapture library were pooled for the exome capture. Pooled libraries were concentrated using the Savant SpeedVac Vacuum concentrator (Thermo Fisher Scientific, Mumbai, India) and adapter-to-adapter hybridization was blocked using the IDT xGen Universal Blockers (IDT). The exonic regions were subsequently hybridized and captured using the xGen Exome Hyb Panel v2 kit (IDT), followed by postcapture amplification and clean up. Qualitative and quantitative assessment of the prepared library was done using automated electrophoresis on the TapeStation system (Agilent, Manesar, India) and fluorometric quantification on the Qubit fluorometer (Thermo Fisher Scientific). Finally, the prepared WES library was sequenced at 100× average coverage generating 150 base-paired end reads on the Illumina NovaSeq 6000 platform.

### Germline Variant Analysis and Interpretation

Alignment, postprocessing, and default quality-filtered variant calling were conducted using the DRAGEN Bio-IT platform, developed by Illumina Inc. The analysis was facilitated by the Dynamic Read Analysis for GENomics (DRAGEN) v4.2.4 pipeline, using the GRCh38 (hg38) human reference genome. Variant annotation and classification were done using VarSeq software (Golden Helix Inc, Bozeman, MT). Variants in the 19 genes included in the National Comprehensive Cancer Network (NCCN) guidelines for PCa testing were assessed,^28^ specifically *ATM*, *BARD1*, *BRACA1*, *BRCA2*, *BRIP1*, *CDH1*, *CHEK2*, *EPCAM*, *HOXB13*, *MLH1*, *MSH2*, *MSH6*, *PALB2*, *PMS2*, *PTEN*, *RAD51C*, *RAD51D*, *STK11*, and *TP53*. Variants were classified using ACMG (American College of Medical Genetics, Bethesda, MD) criteria for determining pathogenic or likely pathogenic variants.

### Statistical Analysis

Categorical and continuous variables extracted from demographic and clinical data were described using frequencies and central tendencies. Similarly, absolute counts and percentages were used to describe variant frequency. Associations between clinical characteristics and the presence of PV/LPVs were determined using chi-square testing and the Kruskal-Wallis test for continuous variables. All analyses were done using SPSS v29 (IBM, New York, NY) and R version 3.6.1. The distributions of variant frequencies for each gene were visualized using a lollipop plot supported by a mutation plotter (cBioPortal, Center for Molecular Oncology at Memorial Sloan Kettering).

## RESULTS

### Baseline Characteristics

A total of 276 patients diagnosed with metastatic PCa were invited to participate in this study. The median age at diagnosis was 65 years (IQR 58-71; Table 2). The median PSA level at diagnosis was 86.44 ng/mL (IQR, 26.02-276.20), and two thirds (184/276) of biopsy specimens included tumors with grade group 4 or 5 disease (score ≥8). On the basis of established risk and volume parameters, 142 patients (51.4%) had high-risk disease and 150 patients (54.3%) had high-volume disease (Table 2).

**TABLE 2.** Patient Characteristics

Characteristics	Total Cohort (N = 276)
Age, years, No. (%)	
<50	24 (8.7)
≥50	252 (91.3)
Baseline PSA (ng/nL), median (IQR)	96.85 (33.4-275.4)
NA	12 (4.3)
Gleason score, No. (%)	
<8	67 (24.3)
≥8	184 (66.7)
NA	25 (9.1)
Resistance status, No. (%)	
Castrate-sensitive PCa	174 (63.0)
Castrate-resistant PCa	101 (36.6)
NA	1 (4.0)
Risk, No. (%)	
High	151 (54.7)
Low	104 (37.7)
NA	21 (7.6)
Volume, No. (%)	
High	151 (54.7)
Low	104 (37.7)
NA	21 (7.6)
Liver metastasis, No. (%)	20 (7.2)

Abbreviations: NA, not available; PCa, prostate cancer; PSA, prostate-specific antigen.

### Detected Variants

**FIG 1.**
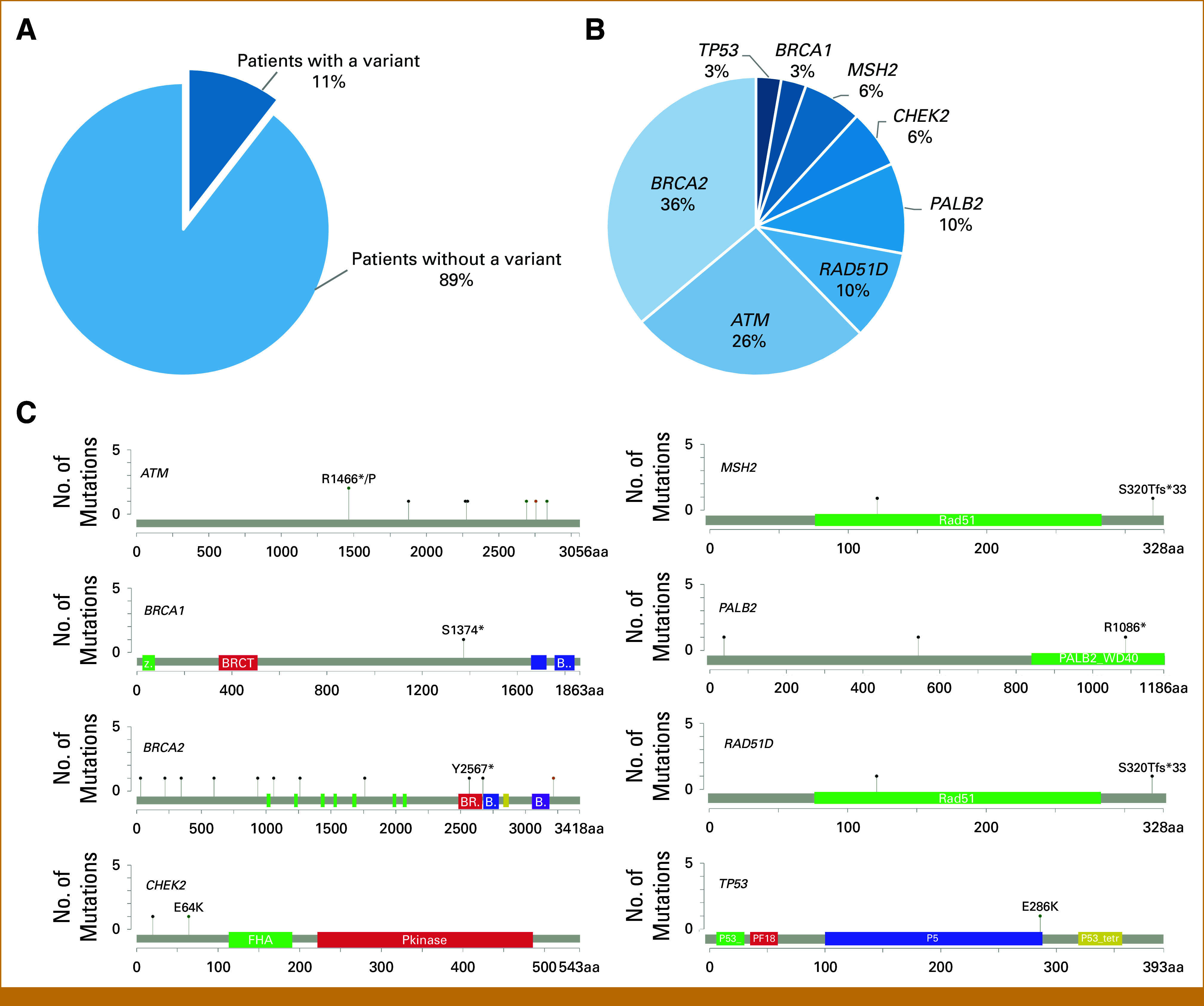
(A) Prevalence of detected PV/LPVs related to PCa in a cohort of 276 individuals with metastatic PCa from India. (B) Distribution of HRR variant frequencies detected across all positive patients (n = 29). (C) Lollipop diagrams reflecting distribution of specific variants of each gene across their respective protein sequences. Functional protein domains are highlighted by colored boxes (cBioPortal). HRR, homologous recombination repair; PCa, prostate cancer; PV/LPV, pathogenic and likely pathogenic variant.

Among 276 patients tested, 29 (10.5%) carried a PV/LPV in at least one of the genes assessed (Fig 1A). Variants were identified across eight genes (*ATM*, *BRCA1*, *BRCA2*, *CHEK2*, *MSH2*, *PALB2*, *RAD51D*, and *TP53*) and most frequently identified in *BRCA2* (3.98%) followed by *ATM* (2.89%; Fig 1B). In total, 31 variants across eight genes were identified in 29 patients (Table 3), with two patients each carrying two distinct mutations. Variants were mapped to analogous amino acid positions along respective protein sequences (Fig 1C). No variants were detected more than once, although two in *ATM* affected the same amino acid (R1466).

**TABLE 3.** Variant Details Detected in the Cohort by Gene

Markers	Gene Names	Transcript Name (clinically relevant)	Exon Number (clinically relevant)	HGVS c. (clinically relevant)	HGVS p. (clinically relevant)	Sequence Ontology (clinically relevant)	Carrier Count
2:47410334-G-T	MSH2	NM_000251.3	3	NM_000251.3:c.607G>T	NP_000242.1:p.Gly203Ter	stop_gained	1
2:47475180-C-T	MSH2	NM_000251.3	12	NM_000251.3:c.1915C>T	NP_000242.1:p.His639Tyr	missense_variant	1
3:48464039-A-G	ATRIP	NM_130384.3	10	NM_130384.3:c.1883-2A>G	?	splice_acceptor_variant	1
11:108289761-C-T	ATM	NM_000051.4	29	NM_000051.4:c.4396C>T	NP_000042.3:p.Arg1466Ter	stop_gained	1
11:108289762-G-C	ATM	NM_000051.4	29	NM_000051.4:c.4397G>C	NP_000042.3:p.Arg1466Pro	missense_variant	1
11:108304809-DelCTCG	ATM	NM_000051.4	37	NM_000051.4:c.5632_5635delTCGC	NP_000042.3:p.Ser1878Lysfs*38	frameshift_variant	1
11:108325542-C-T	ATM	NM_000051.4	46	NM_000051.4:c.6805C>T	NP_000042.3:p.Gln2269Ter	stop_gained	1
11:108326100-DelG	ATM	NM_000051.4	47	NM_000051.4:c.6850delG	NP_000042.3:p.Val2284Leufs*26	frameshift_variant	1
11:108335029-C-T	ATM	NM_000051.4	55	NM_000051.4:c.8071C>T	NP_000042.3:p.Arg2691Cys	missense_variant	1
11:108335962-G-A	ATM	NM_000051.4	56	NM_000051.4:c.8268+1G>A	?	splice_donor_variant	1
11:108345818-C-T	ATM	NM_000051.4	58	NM_000051.4:c.8494C>T	NP_000042.3:p.Arg2832Cys	missense_variant	1
13:32319101-G-A	BRCA2	NM_000059.4	3	NM_000059.4:c.92G>A	NP_000050.3:p.Trp31Ter	stop_gained	1
13:32329468-DelTG	BRCA2	NM_000059.4	8	NM_000059.4:c.658_659delGT	NP_000050.3:p.Val220Ilefs*4	frameshift_variant	1
13:32332511-DelA	BRCA2	NM_000059.4	10	NM_000059.4:c.1037delA	NP_000050.3:p.Asn346Thrfs*3	frameshift_variant	1
13:32333272-DelATCTT	BRCA2	NM_000059.4	10	NM_000059.4:c.1796_1800delCTTAT	NP_000050.3:p.Ser599Ter	frameshift_variant	1
13:32337161-DelAAAC	BRCA2	NM_000059.4	11	NM_000059.4:c.2808_2811delACAA	NP_000050.3:p.Ala938Profs*21	frameshift_variant	1
13:32337531-DelTG	BRCA2	NM_000059.4	11	NM_000059.4:c.3176_3177delTG	NP_000050.3:p.Leu1059Glnfs*7	frameshift_variant	1
13:32338140-C-A	BRCA2	NM_000059.4	11	NM_000059.4:c.3785C>A	NP_000050.3:p.Ser1262Ter	stop_gained	1
13:32339634-C-G	BRCA2	NM_000059.4	11	NM_000059.4:c.5279C>G	NP_000050.3:p.Ser1760Ter	stop_gained	1
13:32357825-T-G	BRCA2	NM_000059.4	16	NM_000059.4:c.7701T>G	NP_000050.3:p.Tyr2567Ter	stop_gained	1
13:32363218-DelAA	BRCA2	NM_000059.4	18	NM_000059.4:c.8020_8021delAA	NP_000050.3:p.Lys2674Aspfs*6	frameshift_variant	1
13:32398161-G-A	BRCA2	NM_000059.4	27	NM_000059.4:c.9649-1G>A		splice_acceptor_variant	1
16:23607958-G-A	PALB2	NM_024675.4	12	NM_024675.4:c.3256C>T	NP_078951.2:p.Arg1086Ter	stop_gained	1
16:23634913-C-A	PALB2	NM_024675.4	4	NM_024675.4:c.1633G>T	NP_078951.2:p.Glu545Ter	stop_gained	1
16:23637952-DelG	PALB2	NM_024675.4	3	NM_024675.4:c.109delC	NP_078951.2:p.Arg37Valfs*16	frameshift_variant	1
17:7673764-C-T	TP53	NM_000546.6	8	NM_000546.6:c.856G>A	NP_000537.3:p.Glu286Lys	missense_variant	1
17:35100981-InsCTCTG	RAD51D	NM_002878.4	10	NM_002878.4:c.955_959dupCAGAG	NP_002869.3:p.Ala321Argfs	frameshift_variant	2
17:35107105-DelT	RAD51D	NM_002878.4	5	NM_002878.4:c.363delA	NP_002869.3:p.Ala122Glnfs*14	frameshift_variant	1
17:43091008-DelCT	BRCA1	NM_007294.4	11	NM_007294.4:c.4120_4121delAG	NP_009225.1:p.Ser1374Ter	frameshift_variant	1
22:28734532-C-T	CHEK2	NM_007194.4	2	NM_007194.4:c.190G>A	NP_009125.1:p.Glu64Lys	missense_variant	1
22:28734664-G-A	CHEK2	NM_007194.4	2	NM_007194.4:c.58C>T	NP_009125.1:p.Gln20Ter	stop_gained	1

### Association Between Patient Characteristics and Variants

Patient features were assessed (age, risk, volume, or presence of liver metastasis) for associations with PV/LPV status. No significant independent associations were observed between those with detectable variants and those without (Table 4). Given that individual genes may elicit distinct clinical features, a secondary exploratory analysis was done to determine whether *BRCA2*, having the most frequent variations, was associated with any patient features compared to other genes (Data Supplement, Table S2). A subtle trend toward lower PSA concentrations in *BRCA2* carriers compared with those without detectable variants was noticed, but not statistically significant.

**TABLE 4.** Comparison of Patient Characteristics by Variant Status

Characteristic	PV/LPV (n = 29)	No Variant (n = 247)	*P*
Age, years, No. (%)			.739
<50	3 (10.3)	21 (8.5)	
≥50	26 (89.7)	226 (91.5)	
Baseline PSA (ng/nL), median (IQR)	119 (21.7-519.50)	85.21 (26.9-273.7)	.44
Gleason score, No. (%)			.924
<8	7 (24.1)	60 (24.1)	
≥8	20 (69)	164 (66.4)	
NA	2 (6.9)	23 (9.3)	
Resistance status, No. (%)			.173
Castrate-sensitive PCa	15 (51.7)	159 (64.6)	
Castrate-resistant PCa	14 (45.2)	87 (35.7)	
NA	1 (0.0)		
Risk, No. (%)			.405
High	18 (62)	133 (53.8)	
Low	9 (31)	95 (38.5)	
NA	2 (7)	19 (7.7)	
Volume, No. (%)			.410
High	13 (44.8)	137 (55.5)	
Low	14 (48.3)	91 (36.8)	
NA	2 (6.9) *0*	19 (7.7)	
Liver metastasis, No. (%)			.919
Absent	25 (92.6)	208 (92.0)	
Present	2 (7.4)	18 (7.3)	

Abbreviation: NA, not available; PCa, prostate cancer; PSA, prostate-specific antigen; PV/LPV, pathogenic and likely pathogenic variant.

## DISCUSSION

The importance of genetic predisposition to high-risk and metastatic PCa is becoming increasingly recognized. The results from this study further substantiate these observations by showing similar frequencies of PCa-related PV/LPVs carried by a group of men with metastatic PCa based out of a large tertiary care center in India. The overall number of currently classified PV/LPVs observed was similar to those identified in other global cohorts, including those reported by Pritchard et al.^9^

Deficits in the HRR pathway lead to an accumulation of genetic mutations in tumors that are related to metastatic potential and variants in these genes are associated with an increased risk of aggressive PCa. This was first observed in a predominantly Caucasian cohort (Pritchard et al),^9^ where 11.8% of patients carried an HRR variant. Since then, findings have been reported from cohorts around the world, with variant detection frequencies ranging from 2% to 18.3% (Data Supplement, Table S1). These trends were reinforced by this Indian cohort, as one in 10 individuals carried at least one PV/LPV in a PCa-related gene, adding to the global literature regarding rates of PV/LPV in metastatic PCa.

Importantly, *BRCA2* variants were the most frequently detected in this study, reflecting the importance of this gene in high-risk PCa. However, not all studies have detected a high-rate of *BRCA2* variants,^4,5^ making it necessary to consider additional pathogenic variants (Data Supplement, Table S1). Indeed, the pattern of variant frequencies observed in this study are distinct to others, indicating population-specific trends. For example, although *BRCA2* variants were most frequently detected in this study and Pritchard's study, the Pritchard group found relatively higher variant frequencies in *CHEK2* and *BRCA1*, whereas we detected relatively higher variant frequencies in *PALB2*, *ATM*, and *RAD51D*. Similarly, we detected relatively more *ATM* variants overall compared with other reviewed cohorts.

Differences in reported frequencies (Data Supplement, Table S1) may be due to a variety of factors. HRR variants are associated with distinct pathogenicity and clinical features, which have been reviewed elsewhere,^29^ suggesting that inclusion criteria may enrich for certain variants. For example, we detected the highest frequency of ATM variants, but this may be due to its association with aggressive disease^29^ and the fact that our cohort reflected only individuals with metastatic disease. Comparatively, some of these differences may be attributable to population composition, for example, founder mutations in *CHEK2* have been identified in those with Ashkenazi Jewish ancestry,^30^ so detection of *CHEK2* variants may be higher in cohorts made up of relatively more individuals with that ancestry.

Genetic variants also play a role in disease progression and management. Some research has shown that PV/LPV frequencies are related to higher Gleason scores,^5,9^ although these findings were not replicated in this study or by Nicolosi et al.^10^ *BRCA2* variants have also been associated with poor prognosis in metastatic PCa, independent of Gleason score^2,31,32^; however, this was also not observed in this study. It remains unclear whether having any or a specific PV/LPV potentiates negative outcomes, which is likely because of limitations in cohort sizes and available clinical data.

Documented germline variant frequencies also play an important role in disease screening and early detection. Individuals suspicious of carrying germline variants, on the basis of guidelines like those provided by NCCN, should be offered genetic testing using panels that incorporate the most up-to-date PV/LPVs. For individuals with active disease, these findings may support clinical management decisions. In cases with germline variants, traceback testing provides an opportunity to identify relatives who may benefit from preventative care. Of note, age of onset has not been strongly associated with germline variants in metastatic PCa, shown here and by others,^9,10^ suggesting that the decision for testing should not be age-independent.

To our knowledge, the findings from this study mark the first of its kind using a prospective cohort of patients with metastatic PCa from India. The WES strategy allows for broad exploration of germline variants carried by individuals within this cohort and confirms the association of PCa-related variants in metastatic PCa. However, the single-center design, focusing on patients from a tertiary care center in northern India, may not adequately reflect the large and diverse Indian population. Future analysis that integrates this cohort into a larger data set that includes those from other centers will provide additional insights to germline variants in this context. Furthermore, collection of race/ethnicity and family history may help to identify differences in variant frequencies among subpopulations. Future work will be done to look at prevalence of variants of unknown significance or any unique variants specific to this cohort that may be of interest.

To conclude, in this single-center study from India, in patients with metastatic PCa, we found a high prevalence of germline P/LP mutations in PCa-related genes, which is similar to frequencies detected in other populations. Also, the association between variants with clinical and outcome variables remains challenging because of the number of variants detected overall as well as their varying functional effects.
